# Enhancing reading performance through action video games: the role of visual attention span

**DOI:** 10.1038/s41598-017-15119-9

**Published:** 2017-11-06

**Authors:** A. Antzaka, M. Lallier, S. Meyer, J. Diard, M. Carreiras, S. Valdois

**Affiliations:** 1 Basque Center on Cognition, Brain and Language, 20009 San Sebastián, Spain; 2 Departamento de Lengua Vasca y Comunicación, UPV/EHU, 48940 Leioa, Spain; 3 Université Grenoble-Alpes, LPNC, F-38040 Grenoble, France; 4 CNRS, LPNC UMR5105, F-38040 Grenoble, France; 5 Ikerbasque, Basque Foundation for Science, 48013 Bilbao, Spain

## Abstract

Recent studies reported that Action Video Game-AVG training improves not only certain attentional components, but also reading fluency in children with dyslexia. We aimed to investigate the shared attentional components of AVG playing and reading, by studying whether the Visual Attention (VA) span, a component of visual attention that has previously been linked to both reading development and dyslexia, is improved in frequent players of AVGs. Thirty-six French fluent adult readers, matched on chronological age and text reading proficiency, composed two groups: frequent AVG players and non-players. Participants performed behavioural tasks measuring the VA span, and a challenging reading task (reading of briefly presented pseudo-words). AVG players performed better on both tasks and performance on these tasks was correlated. These results further support the transfer of the attentional benefits of playing AVGs to reading, and indicate that the VA span could be a core component mediating this transfer. The correlation between VA span and pseudo-word reading also supports the involvement of VA span even in adult reading. Future studies could combine VA span training with defining features of AVGs, in order to build a new generation of remediation software.

## Introduction

Action video games (AVGs) and books are very different and yet, the visual processes involved in playing AVGs and in reading a book could be closely linked. The former display complex scenes, sophisticated geometric rendering and rapid moving objects, whereas the latter contain black and white static print, usually in a single alphabet and font. However, Franceschini *et al*.^1,2^ showed a positive effect of AVG training on reading in dyslexic children (see also Gori *et al*.^3^). A group of children played AVGs for twelve hours. After training, they significantly improved both their visual attention and their reading speed, without loss of reading accuracy. This observation is puzzling. How could an off-the shelf AVG have such an impact on reading performance? Could AVGs be used to improve reading speed, without specifically targeting verbal material?

There are several possible links between AVG playing and reading. AVGs are defined by high speed events and fast moving targets, spatial and temporal unpredictability, an emphasis on the peripheral visual field, and high motor, perceptual and cognitive loads^4^. They are one of the most studied classes of video games because of their positive effect on various cognitive and perceptual processes. For example, AVG players are better in contrast discrimination^5^, probabilistic inference^6^ and mental rotation tasks^7^. The features of AVGs set up high attentional requirements to succeed at the game, so we could assume that playing AVGs specifically trains visual attention. And indeed, it does: the causal link between AVG practice and improvement of visual attention has been assessed in many studies^8,9^ (for a review see ref.^4^). Visual attention covers a large number of dimensions, so that AVGs affect many different tasks. To name a few, AVG players are better at visual search^10,11^, in enumeration tasks and in multiple-object-tracking tasks^9^. They also are less susceptible to crowding effects^12^ (for a review see ref.^13^). Interestingly, individual improvement in reading performance was correlated with improvement in visual attention in the studies by Franceschini *et al*.^1,2^.

Temporal and spatial visual attention is enhanced in AVG players. Their higher performance on tests of visuo-attentional skills (enumeration, multiple-object-tracking) was attributed to spatial attention improvement^13^. This is in particular exemplified by their ability to track more objects simultaneously than non players^14^. Other findings suggest positive effects on the temporal dimension of visual attention. This is in particular exemplified in the attentional blink paradigm in which a stream of letters is briefly displayed, one after the other, and participants have to quickly shift from one letter feature (the colour of a first target) to another (the identity of a second target). The momentary blink in attention, observed when the second target occurs in the few hundred milliseconds following the coloured letter, is reduced in AVG players, suggesting faster temporal processing skills than in non players^8^. It is thus well established that AVGs improve several facets of attention and that AVG players benefit from greater attentional resources.

On the other hand, many studies have shown that visuo-attentional skills are involved in normal^15,16^ and pathological reading^17,18^. Our previous findings, in particular, emphasized the link between the visual attention (VA) span and reading performance^19–23^. VA span is defined as the number of distinct visual elements (i.e., letters, in a reading context) that can be simultaneously processed in one fixation^24^. The size of the VA span reflects the amount of attention capacity that is available for multi-element processing^25,26^ and is linked to superior parietal lobule (SPL) activation for pre-lexical orthographic processing^27–29^. In typical children/teenagers, a larger VA span relates to faster and more accurate reading^19,20^. A large VA span helps readers to process larger orthographic units^21,30^. Individuals with a large VA span can process most familiar words and within-unfamiliar-words longer sub-lexical units (multi-letter graphemes or syllables) as a whole, which results in higher reading speed. Thus, children with higher VA span process more letters at each fixation and show faster text reading^31^. The impact of VA span on text reading further holds for adult readers^32^. The contribution of VA span to pseudo-word reading is independent of phonological skills in typical readers^19^ and a subset of dyslexic children exhibits a VA span deficit and poor pseudo-word reading despite good phonological skills^22–24,33^.

Some authors argue that VA span impairment would be the consequence of the poor reading skills of dyslexic readers^34^ but evidence from longitudinal and training studies speaks against such a consequence link^35^. The VA span of pre-readers predicts their future reading performance^36^. In pure visual attention span dyslexia, VA span training has a significant impact on reading speed^37,38^. The fact that the VA span is involved in non-verbal tasks and non-verbal material^25,28,39^ is further evidence against any interpretation that the VA span-reading relationship is mediated by language or reading experience. Furthermore, the VA span deficit in developmental dyslexia is not restricted to horizontal reading-like displays. Children showing VA span limitations on horizontal letter strings are similarly impaired when using circular displays^26,40^. They further show a deficit in visual search tasks for spatially distributed stimuli, which strongly speaks against specialization for reading and/or horizontal array processing^41^. Because the VA span is not restricted to verbal stimuli, and because it is not restricted to mono-dimensional stimuli, it could also be used by players when processing visual stimuli presented by an AVG.

Overall, AVG playing affects the spatial distribution of attention over the visual scene so that a larger deployment of attentional resources helps AVG players to process more visual information simultaneously. On the other hand, children with higher VA span have greater attentional resources which they deploy more widely to process more letters simultaneously within strings, leading in turn to faster and more accurate reading. When put together, these results strongly suggest that the VA span might be a common component between playing AVGs and reading. AVG playing might enlarge the players’ VA span, which would help them process larger multi-letter units, and consequently, would improve their reading performance. The main aim of the current study is to test this hypothesis. Two groups of AVG players and non-video game (NVG hereafter) players were recruited; they were matched on chronological age and text reading proficiency. We assessed their VA span abilities (i.e., their ability to process multiple elements simultaneously) using classical partial and global report paradigms. During partial (or global) report, a 6-consonant string is briefly presented to the participant. One string position (or none) is cued at each trial, and the participant has to name the cued letter only (or the whole sequence). Single letter identification skills were further assessed to ensure that differences in performance on the letter-string report tasks were not explained by differences in single letter processing. Finally, we tested the participants’ ability to read briefly presented 6-letter pseudo-words (PW) made of units of different sizes. AVG players were expected to show larger VA span and more accurate PW reading; they should also be more sensitive to larger PW sub-lexical units than non-players.

## Results

### Visual Attention Span Tasks

#### Global Report

**Figure 1:**
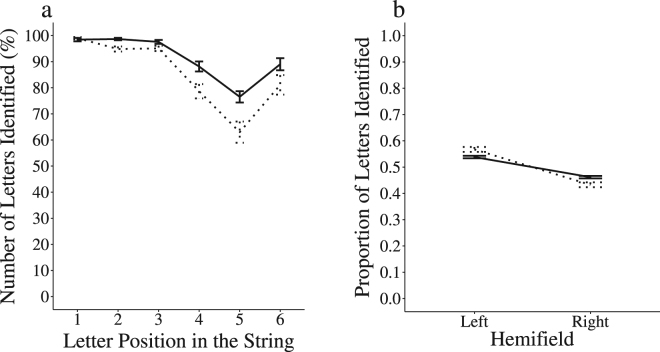
Global report task - Letter identification by position (**1a**) and left-right hemifield relative performance (**1b**) for the AVG players () and non-players (). Error bars represent one standard error.

Performance of the two groups of participants in global report is illustrated in Fig. 1 and descriptive data is provided in Supplementary Table S1.

In the global report task, participants were asked to report as many letters as possible from 24 6-letter strings presented successively at the centre of the screen for 200 ms. The percentage of letters accurately identified by position in the AVG (*n* = 19) and NVG (*n* = 17) groups is illustrated in Fig. 1a. A Type III ANOVA was performed on the original data with Group (AVG vs. NVG) as the between-subject factor and Letter Position as the within-subject factor (Positions 1–6). There was a main effect of Group (*F*(1, 34) = 11.35, *p* = 0.002, η_p_^2^ = 0.25) and a main effect of Letter Position (*F*(5, 170) = 24.38, *p* < 0.001, η_p_^2^ = 0.68). The AVG group identified more letters accurately than the NVG group (M*(SD)* = 91.41 *(4.41)* % vs. M*(SD)* = 85.29 *(6.4)* %). The Group by Letter Position interaction was significant (*F*(5, 170) = 4.04, *p* = 0.002, η_p_^2^ = 0.45). Post hoc comparisons on the Group by Letter Position interaction indicated that the AVG and NVG groups performed similarly on the three first positions of the string (Positions 1, 2 and 3; all *ps* > 0.19), but the AVG group identified more letters accurately on Position 4 (β = 9.48, *t* = 3.2, *p* = 0.002), Position 5 (β = 13.54, *t* = 4.57, *p* < 0.001) and Position 6 (β = 7.91, *t* = 2.67, *p* = 0.008).

We reasoned that better parallel processing in AVG players would result in a more homogeneous spreading of visual attention across the whole string, thus resulting in more balanced identification of letters across the hemifields. To test this hypothesis, a left-right hemifield comparison was performed while equating for the two groups overall performance on the task. The percent average accuracy of participants to the left (positions 1, 2, and 3; left hemifield) and right of fixation (across positions 4, 5, and 6; right hemifield) were computed. These scores were then divided by the participant’s mean percent score, across all positions. Thus, the sum of both scores for each participant was 1. Therefore, the “Hemifield” scores represented the relative performance on each hemifield for each participant, regardless of their overall accuracy, thus reflecting their visual attention distribution strategy over the string. Hemifield effects on global report performance are illustrated in Fig. 1b. A Type III ANOVA was performed on the original data with Group (AVG vs. NVG) as the between-subject factor and Hemifield as the within-subject factor. As expected, there was no main effect of Group (*F*(1, 34) = 0, *p* = 1, η_p_^2^ = 0) due to the normalized scores used (see above). There was a main effect of Hemifield (*F*(1, 34) = 29.96, *p* < 0.001, η_p_^2^ = 0.47) and a Group by Hemifield interaction (*F*(1, 34) = 8.11, *p* = 0.007, η_p_^2^ = 0.19). The post hoc comparisons on the Group by Hemifield interaction indicated that in both Groups, participants responded more accurately to letters presented in the left as compared to the right hemifield (AVG: β = 0.08, *t* = 7.74, *p* < 0.001; NVG: β = 0.13, *t* = 12.87, *p* < 0.001). Moreover, the NVG participants had a relative performance score above that of the AVG participants on the left hemifield (AVG-NVG: β = −0.03, *t* = −2.85, *p* = 0.006) but below that of the AVG participants on the right hemifield (AVG-NVG: β = 0.03, *t* = 2.85, *p* = 0.006). This suggests a stronger left bias for the NVG than the AVG participants.

If more widely spread across the letter string, higher attentional resources would further result in lesser inter-position variability on letter identification. The correlation between individual standard deviation scores (reflecting individual inter-position variability in performance on the global report task) and individual mean percent scores across all positions on the global report task was significant both across the whole group of participants (*n* = 36, τ_b_ = −0.77, *p* < 0.001), and within each group of participants (AVG (*n* = 19): τ_b_ = −0.78, *p* < 0.001; NVG (*n* = 17): τ_b_ = −0.73, *p* < 0.001, see Supplementary Fig. S1). This indicates that smaller variability related to better overall performance on the tasks. In particular, the AVG group showed lower by-position variability in performance than the NVG group (AVG: *M(SD*) = 8.9 *(3.29)*, NVG: *M(SD)* = 13.81 *(5.98)*, *t* = −3.01, *df* = 24.25, *p* = 0.006).

#### Partial Report

**Figure 2:**
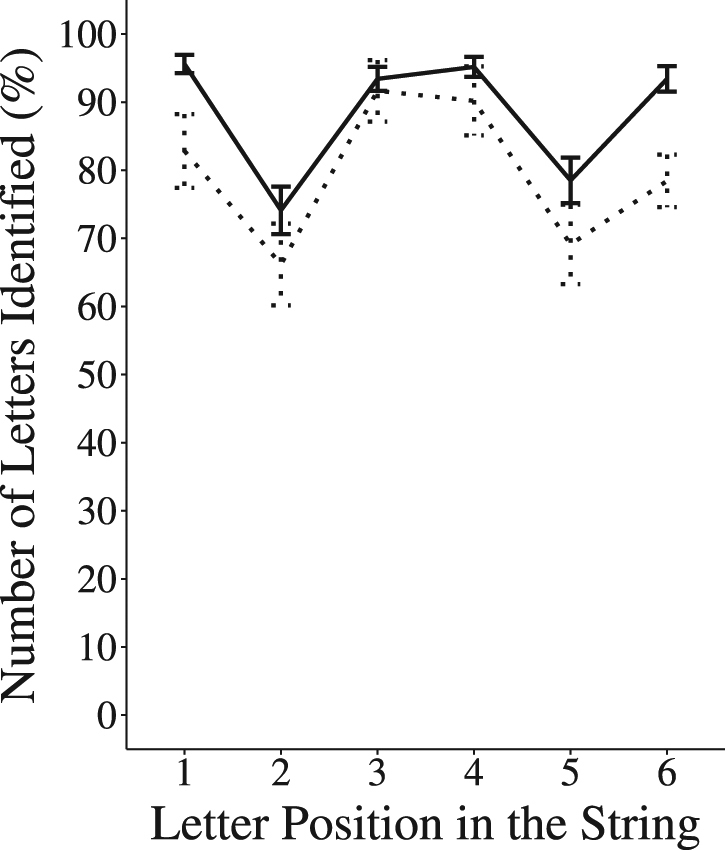
Partial report task - Letter identification by position for the AVG players () and non-players (). Error bars represent one standard error.

In the partial report task, the participants were successively presented 72 6-letter strings briefly (200 ms) in the centre of the screen. After the presentation of each string a retro-cue indicated which letter they should report from the presented string. The percentage of cued letters accurately identified by position in the AVG (*n* = 19) and NVG (*n* = 17) groups is illustrated in Fig. 2 and descriptive data is provided in Supplementary Table S2. A Type III ANOVA was performed on the original data with Group (AVG vs. NVG) as a between-subject factor and Letter Position as a within-subject factor (Positions 1–6). There was a main effect of Group (*F*(1, 34) = 6.28, *p* = 0.017, η_p_^2^ = 0.16) and a main effect of Letter Position (*F*(5, 170) = 8.70, *p* < 0.001, η_p_^2^ = 0.55). As in global report, the AVG Group identified significantly more letters than the NVG Group (M*(SD)* = 88.38 *(5.95)* % vs. M*(SD)* = 79.74 *(13.66)* %) but the Group by Letter Position interaction was not significant (*F*(5, 170) = 1.09, *p* = 0.36, η_p_^2^ = 0.27), meaning that the AVG players’ advantage was similar across the six positions.

The correlation between individual standard deviation scores (reflecting individual inter-position variability in performance on the partial report task) with individual mean percent scores on the partial report task was significant both across the whole group of participants (*n* = 36, τ_b_ = −0.73, *p* < 0.001), and within each group of participants (AVG(*n* = 19): τ_b_ = −0.62, *p* < 0.001; NVG(*n* = 17): τ_b_ = −0.77, *p* < 0.001, see Supplementary Fig. S2). Once again, as expected, based on the higher mean performance of the AVG as compared to the NVG group, the former also showed lower by-position variability in performance (AVG: *M(SD)* = 10.85 *(5.13)*, NVG: *M(SD)* = 16.71 *(8.38)*, *U* = 93.5, *Z* = −2.16, *p* = 0.030, *r* = −0.36).

#### Control Task

The single letter (SL) identification task was used as a control to ensure that the AVG and NVG groups did not differ on single letter processing, so that better performance on global and partial report in the AVG group could be reliably interpreted as evidence of higher multi-letter processing skills. Group comparison revealed no difference between the AVG players and non-players (*U* = 157.5, *Z* = −0.18, *p* = 0.88, *r* = −0.03) on their computed threshold (reflecting the shortest presentation duration at which at least 80% letters were accurately identified) on this task (AVG: *n* = 19, *M(SD)* = 36.58 *(7.12)*, NVG: *n* = 17, *M(SD)* = 37.00 *(7.43)*). As further evidence for the independence of performance in single and multi-letter processing, a weighted sum of performance on the SL task (score at 33 ms * 5+ score at 50 ms * 4+ score at 67 ms * 3+ score at 84 ms * 2+ score at 101 ms^32^) was used to correlate with performance on the global and partial report tasks. The correlations were not significant (*n* = 36, GL:τ_b_ = 0.21, *p* = 0.10, PR: τ_b_ = 0.03, *p* = 0.82) suggesting that the differences between the two groups were not related to single letter processing, but are apparent only when multi-letter processing is required.

### Pseudo-word Reading

The AVG and NVG players were asked to read aloud pseudo-words that were briefly presented at the centre of the computer screen for 60 ms and followed by a mask. All pseudo-words were 6-letters long but they varied in the number of syllables, either including three CV syllables (CVCVCV pseudo-words such as “siluve”) or two syllables. In the latter case, the second syllable included a long vocalic grapheme (CVCVlg as in “rig**ois**”). Half of the pseudo-words of each syllable-length included an existing word corresponding to either a CVCV word for the 3 syllable-long pseudo-words (e.g., “ri**mode**”), or a CVlg word for the 2-syllable long pseudo-words (e.g., “gi**bois**”). The number of pseudo-words accurately named by the AVG and NVG players is provided on Table 1.

**Table 1:** Accuracy of the AVG and NVG group in pseudo-word (PW) reading.

Group		CVCVCV PWs		CVCVlg PWs	
		Word Present	Word Absent	Word Present	Word Absent
AVG (*n* = 19)	Mean (SD)	0.83*(0.17)*	0.77*(0.16)*	0.79*(0.16)*	0.78*(0.17)*
	Range	0.24−1	0.25−0.95	0.29−1	0.29−1
NVG (*n* = 17)	Mean (SD)	0.73*(0.17)*	0.67*(0.19)*	0.68*(0.18)*	0.66*(0.15)*
	Range	0.33−1	0.30−0.9	0.14−0.86	0.38−0.9

The generalized linear mixed effects model used to analyse the accuracy of responses on each trial included the fixed effects of Group (AVG vs. NVG), Word Presence (Word Present vs. Word Absent) and Grapheme Size (presence of a large grapheme or not) and their interactions. All factors were coded as sum contrasts. The most complex random effect structure that converged included random intercepts by subject and item and a random by subject slope for Grapheme Size. Information on the model is provided in Supplementary Table S3. The exclusion of two outliers (one from the NVG and one from the AVG group) did not change the pattern of results and significance so all participants were included in the analysis.

Only the effect of Group was significant with the AVG group naming 79.34% (*SD* = 14.87) pseudo-words accurately against 68.44% (*SD* = 15.42) for the NVG group (β = −0.37, *z* = −2.39, *p* = *0.017*). No effect of pseudo-word structure/grapheme size (CVCVCV vs. CVCVlg) or word presence within the pseudo-word was found (*ps* > 0.19), suggesting that neither the AVG nor NVG group was sensitive to the lexical units embedded within the pseudo-words.

**Figure 3:**
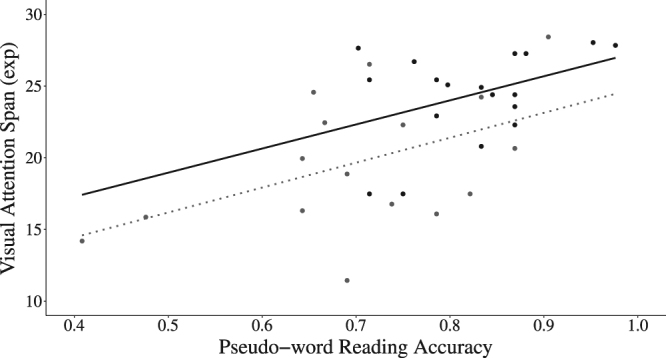
Correlations between a composite measure of VA span and pseudo-word reading accuracy for the AVG players () and non-players ().

Correlations were computed between VA span scores (computed from performance on the global and partial report reduced to the mean number of letters accurately processed in each trial^32^) and pseudo-word reading accuracy. The two outliers of the pseudo-word reading task were excluded and VA span scores were exponentially transformed to improve their distribution. The correlation was significant both within each group (AVG (*n* = 18): *r* = 0.41, *p* = 0.047; NVG (*n* = 16): *r* = 0.48, *p* = 0.047; Fig. 3) and across all participants (r(*n* = 34) = 0.60, *p* < 0.001).

In order to test the variance in pseudo-word reading accuracy explained by VA span skills a regression analysis was performed including the data of both groups of participants. Once again, the two outliers on pseudo-word reading accuracy were removed and VA span scores were exponentially transformed. All the dependent variables were centred. A first regression model included only the control variables chronological age and the weighted score of single letter identification. The second regression model included the control variables and VA span scores. The models were compared using a chi-squared test and comparing the multiple and adjusted r-squared values of the two models. The model including only the control variables (*R* ^2^ = 0.08, adjusted *R* ^2^ = 0.02, *F*(2, 31) = 1.39, *p* = 0.26) did not explain significant variance in pseudo-word naming accuracy while the model including the control variables and VA span scores did explain significant variance (*R* ^2^ = 0.37, adjusted *R* ^2^ = 0.31, *F*(3, 30) = 5.90, *p* = 0.003). In line with this result, the model including VA span scores had a significantly higher goodness of fit than the one including only the control variables (χ^2^(1, *n* = 34) = 0.14, *p* < 0.001), and in this model the effect of VA span was significant (*β* = 0.015, *t* = 3.71, *p* < 0.001). Importantly, when the categorical factor Group (AVG vs. NAVG) was added to the first model with the control variables, the model did explain significant variance in pseudo-word naming accuracy (*R* ^2^ = 0.29, adjusted *R* ^2^ = 0.22, *F*(3, 30) = 4.02, *p* = 0.016). Nevertheless, the addition of the VA span to the model (*R* ^2^ = 0.42, adjusted *R* ^2^ = 0.34, *F*(4, 29) = 5.20, *p* = 0.003) still lead to a significant improvement (χ^2^(1, *n* = 34) = 0.07, *p* = 0.011). The effect of VA span was also significant in this final model (β = 0.011, *t* = 2.55, *p* = 0.016), indicating that VA span skills explained additional unique variance in pseudo-word naming accuracy after taking into account both the control variables and the group effect.

## Discussion

The present study tested the hypothesis that VA span would be a critical feature explaining the effects of AVG training on reading performance^1,2^. Previous evidence for a link between AVGs and reading performance was reported in children and developmental dyslexia. We here focused on two groups of young adults who were either AVG players or non-players (NVG). Our first aim was to explore whether AVG players have higher VA span skills than NVG players. Our second aim was to assess whether they performed better in a pseudo-word reading task, thus providing first evidence that the effect of AVGs on reading extended to expert readers. In addition, we explored the relationship between VA span and reading performance to verify whether faster and more accurate reading in these groups of participants was related to larger VA span.

A first key finding of the current study is the larger VA span observed in AVG players compared to non-players. The positive impact of AVG on visual processing and visual attention has been largely documented (for a review see ref.^42^). Individuals who play AVGs improve their visual sensitivity^6^, are less sensitive to visual interference (or crowding^12^) and show enhanced temporal resolution in attentional blink tasks^43^. However, none of these factors can straightforwardly explain the higher performance of AVG players on the VA span tasks. Higher sensitivity to letter details might impact the participants’ ability to process letter strings, but the two groups of AVG players and non-players were matched for letter identification skills. So, AVG players are more efficient in processing letter-strings despite having single letter processing as fast as the non-players. Crowding can also affect letter string processing^44,45^ but between-consonant spacing was increased in global and partial report to avoid crowding effects, so that better performance of the AVG players on these tasks could hardly be just the consequence of lower sensitivity to interference. Lastly, performance on the VA span tasks, where visual elements are simultaneously displayed, has been shown to dissociate from processes involving rapidly serially presented visual stimuli^46^. Instead, a *resource*-based account of the VA span could explain the current link observed between AVG playing and VA span; given that the connection between playing AVGs and benefits in attentional resources is already well established^8,9,42^. Indeed, larger VA spans on the global and the partial report tasks were shown to reflect the allocation of greater attentional resources to multiple stimuli presented at once^26^. In other words, participants with a larger VA span can allocate more attentional resources to each element presented within the string in parallel, thus enhancing the number of letters that can be accurately identified simultaneously.

To explore more in depth whether higher VA span in the AVG players resulted from the ability to allocate more attentional resources across the letter string, the response pattern of the two groups in the global and partial report tasks was analyzed. In global report, results revealed a weaker left bias in the AVG group as compared to the NVG group, as expected following enhanced parallel processing. However, interpreting the results this way may be incorrect as performance was almost at ceiling for the AVG group on the left hemifield. Interpretation of performance in partial report is more straightforward. In the absence of a ceiling effect, AVG players showed an overall better performance than NVG regardless of the letter target position. The overall findings strongly suggest that AVG players can deploy greater attentional resources in parallel across the letter string to facilitate each letter processing and identification.

Different attention distribution strategies depending on task-demands have previously been reported. Although parallel processing is involved in both global and partial report, a leftward bias of covert attention that relates to the direction of reading is only observed in global report^32^. We postulate that the greater amount of attention resources available in the AVG players allowed them to adopt a wider distribution of their VA span resources, which facilitated the identification of the rightward letters whose identification is harder given the inherent nature of the task. In contrast to global report, there is typically no position bias in partial report since the target letter position is indicated by a retro-cue displayed at the offset of the letter string. Higher attentional resources in AVG players thus yielded better processing of the target letters independently of their position in the string. Furthermore, in both global and partial report, higher performance related to lesser inter-position variability in letter identification. These overall findings suggest that AVG players exhibit greater attentional resources than NVG players allowing them to allocate a higher amount of attentional resources to each position of the letter string.

A second evidence supporting a link between AVG playing, VA span and reading, is the group difference observed on pseudo-word reading skills. It is worth noting that the two groups were a priori matched for text reading proficiency. We reasoned that since the young adult AVG players had learned to read prior to playing AVGs, they should have developed expert reading skills independently of AVG playing experience and should have similar reading skills as the NVG players. Nonetheless, AVG playing during adolescence or later may have modified the VA span skills of AVG players, thus their ability to process more letters simultaneously. However, this was not expected to impact the reading of real words, which are processed as a whole^47^ and recruit minimal attention once acquired^48^. In contrast, the amount of attention resources available was expected to impact the processing of unfamiliar words (or pseudo-words), not yet encoded in long-term memory. Better pseudo-word reading performance was thus expected in the AVG group. As pseudo-words are thought to be serially processed while reading^49,50^, we expected the AVG players to process larger sub-units than the NVG participants, if more attentional resources were available. Because participants in both groups were expert readers, we administered a highly demanding pseudo-word reading task where the stimuli were displayed for only 60 ms.

Importantly, the group of AVG players was far better at reading the briefly displayed pseudo-words than the NVG players but was not more sensitive to the pseudo-word sub-units. In both groups, stimuli including a long sub-unit (either an embedded word or a long syllable) were not read better than those that did not include these sub-units. This suggests that the pseudo-words were mainly processed as a whole without decomposing their internal structure. Although this claim seems counterintuitive when considering the classical models of reading (e.g., refs^49,50^), the multitrace memory model^30^ predicts the parallel processing of pseudo-words when they are briefly presented^30^. This model postulates that the deployment of attentional resources across the entire letter string is the first step of the reading process, regardless of the lexical status of the items (words or pseudo-words). Failing to process the letter string as a whole makes the system switch to an analytic mode, characterized by serial processing of sub-units. The model postulates that switching to the analytic from the global mode imposes some cost on processing illustrated by longer processing times. However, such processing cost could hardly occur in 60 ms. The current findings thus suggest that AVG players are more prone to process pseudo-words accurately as a whole than non-players.

Last, we explored whether AVG players with a larger VA span demonstrated more accurate pseudo-word reading. Significant correlations were found, not only in the AVG player group but also in the non-players and in the whole population. Such VA span/reading relationship was previously reported in young adult expert readers^32^, as well as in healthy and dyslexic children^19,24,33^.

Overall, our findings are consistent with Bavelier, Green & Seidenberg’s^51^ proposal that AVGs enhance visual attention skills thus allowing the processing of larger multi-letter units in reading. More specifically, we propose that playing AVGs improves the ability to simultaneously process multiple elements in visual displays. This benefit would increase VA span resources and significantly contribute to boosting reading performance through the capacity of processing more letters in parallel within strings. This is not to say that VA span is the unique facet of visual attention that is enhanced by AVGs and the sole component that relates to reading skills. Franceschini *et al*.^2^ have shown that playing AVGs improves focused visual attention, as well as working memory and visual-to-auditory attentional shift. We just claim here that the VA span is one of the critical features that contribute to explain why AVGs improve reading skills.

Note that current evidence for a parallel processing enhancement in AVG players would require controlling more strictly for other types of visuo-spatially demanding activities in the participants. Indeed, some sports training^52^ and some daily life situations, as car driving^53^, may more specifically affect parallel processing skills.

We acknowledge that the current findings do not establish a causal relationship between AVG practice, VA span and reading. However, our results highlight the VA span as a critical feature improved by the practice of AVGs that trigger faster and more efficient reading. Training studies are needed to establish causality in showing that initially non players trained with AVGs develop a larger VA span and better reading skills than children trained with non-AVGs. We remain however very confident that the relationship is causal. First, previous studies on AVGs have shown that the results of training studies are nearly identical to the results obtained through comparison of groups of previously AVG players and NVG players^6^. In particular, there is no evidence that AVG players are individuals who had improved visual attention skills prior to becoming players. Second, both VA span and reading skills improve in dyslexic children when trained to simultaneously process briefly presented visual multi-elements, be they alphanumeric or not^38^, thus using training programs that share some common features with AVGs. The current findings pave the way for a new generation of training programs that should be developed to improve reading acquisition and remediate developmental dyslexia focusing on VA span training while adopting the defining features of AVGs.

## Methods

### Participants

A total of 38 French, right-handed adults (18–45 years old) with normal or corrected-to-normal vision, were recruited through advertising at the Grenoble Alpes University Campus and the RISC (Relais d’information sur les sciences de la cognition) mailing list. Recruitment targeted two groups of participants: a group that did not play action video games, named the NVG group, and a group of action video game players, called the AVG group. To be included in the latter group, participants had to have played action video games regularly (at least 5 hours a week) during the six months prior to the study. Information regarding language background, handedness and experience playing action video games was acquired based on a questionnaire (see Supplementary Methods S1). The questionnaire was adapted from previous studies^13,54^. Participants’ reading proficiency was assessed using the text reading task of the Eclat-16+ battery^55^. The test required reading a text as quickly and as accurately as possible for 1 minute. The number of words correctly read (total words read-errors) per minute was calculated for each participant.

A total of 36, out of the total 38 young adults who were recruited, complied with the recruitment criteria and participated in the study. Nineteen participants (14 males) who reported playing AVGs regularly (mean hours played per month = 69.94, *SD* = 37.2; range: 20–173 hours, the hours reported by two participants were removed from these descriptive statistics since they were outliers, reporting 360 and 291 hours played per month^1^) were included in the AVG group (mean age = 20.89, *SD* = 2.66, range: 18–26). Seventeen participants (7 males) fell into the NVG category (mean age = 20.76, *SD* = 2.84, range: 18–28) since they reported either no video game practice during the past year (*n* = 13), experience playing non-action video games (*n* = 2) or very limited experience playing action video games (*n* = 2). The two groups of participants did not differ on age (Wilcoxon signed-rank test: *U* = 170.5, *Z* = 0.29, *p* = 0.78, *r* = 0.05). They were matched on text reading proficiency as measured in number of words accurately read per minute (*M(SD)* = 207.47 *(25.52)* vs. *M(SD)* = 198.65 (*21.26*) for the AVG and NVG groups respectively; *t* = 1.13, *p* = 0.27). Informed written consent was obtained from each participant in accordance with the ethic committee guidelines of the Grenoble-Alpes University (CERNI) and each subject was paid for participating. The protocol was approved by the ethics committee for research activities involving humans (N° ID RCB: 2014-A01823–44) and the experiment was performed in accordance with the relevant guidelines and regulations.

^1^Only video games that could clearly be categorised as non-action video games were excluded from this score.

### Visual Attention Span Tasks

Two tasks of global and partial report were used to measure VA span together with a Single letter identification control task.

#### Global report and partial report

The 6-letter strings used in the global and partial report tasks consisted of different combinations of 10 consonants (B, P, T, F, L, M, D, S, R, H) without letter repetition within a string. Letter strings were presented on a white background in black uppercase Arial font (height 7 mm and inter-consonant space 0.57°). There were a total of 24 trials in the global report task and a total of 72 trials in the partial report task.

In both tasks, trials began with a central fixation point (1000 ms) followed by a blank screen (50 ms) and the centrally displayed 6-letter string (200 ms). Following the letter string presentation in the global report task, participants were instructed to verbally report as many letters as possible, regardless of order. In the partial report task, the letter string was followed by a single vertical bar, appearing 1.1° below one of the previously presented letters in the string and cueing the participants to report that letter only. In both tasks the experimenter typed the participants’ response and proceeded to the next trial by button press without giving feedback. Both tasks were preceded by 10 practice trials during which feedback was provided. The order of these tasks was counterbalanced across participants and the SL task was always administered between the global and partial report tasks. In both tasks the dependent measures were: the percentage of accurately reported consonants either overall or by position and the individual standard deviation scores. The latter were used to study individual inter-position variability in performance and whether smaller variability related to better overall performance on the tasks. Individual standard deviation scores were calculated for each participant based on individual scores of percent average performance on Positions 1–6.

#### Single letter identification

In the single letter identification task, trials consisted of presenting each of the consonants used in the global and partial report tasks once for each of five different brief presentation durations (33, 50, 67, 84 and 101 ms) followed by a mask for 150 ms. Participants were asked to name the consonant after presentation. The experimenter typed the participants’ response and proceeded to the next trial by button press. At the beginning of the task, participants were presented with 10 practice trials (2 for each presentation time duration) for which they received feedback. This measure was used as a control to the other two VA span tasks in order to identify whether differences between the two groups could be related to single as opposed to multi-element processing abilities. A threshold was computed based on the shortest presentation duration at which at least 80% of letters were accurately identified.

### Pseudo-word Reading

A Pseudo-word Reading task was created for the purpose of the present study, with the aim to measure the reading skills of participants in particularly challenging situations, and capture the potential effect of video game practice on reading. The stimuli were six-letter pseudo-words pertaining to one of four conditions including 21 stimuli. The conditions differed based on the presence of familiar orthographic chunks embedded within the pseudo-words (e.g., letter clusters representing complex graphemes or words). The four conditions resulted from the orthogonal manipulation of two factors: Word Presence, with either a word present or absent within the pseudo-word and Grapheme Size, with either smaller or larger graphemes present in the pseudo-word (see Supplementary Methods S2). In all conditions, the first two letters of the pseudo-word corresponded to a CV structure and the manipulations were restricted to the remaining four letters. In the Word Present conditions, the initial CV syllable was followed by a familiar string of letters corresponding to a frequent French word (e.g. “*gare”*, station or “*main”*, hand). These letter strings also appear within other words in the French language although no semantic relationship is generally shared between the embedded word and the whole word (e.g., “*ci* ***gare*** *”*, cigar or “*de* ***main*** *”*, tomorrow*)*. Half of the stimuli in the Word Present condition corresponded to the Small Grapheme condition including small grapheme clusters (e.g., “*so* ***gare*** *”*, “*pi* ***mine*** *”*), while the other half corresponded to the Large Grapheme condition, including words composed of at least one larger grapheme cluster (e.g., “*gi* ***m*** ***ain*** *”*, “*fé* ***p*** ***eau*** *”*). In the Word Absent condition, the initial CV syllable was followed by syllable structures that were similar to the Word Present condition, but that did not correspond to words (e.g., “so***rige*** *”*, “*ne* ***lain*** *”*). Once again, half of the stimuli in the Word Absent condition corresponded to the Small Grapheme condition (e.g., “so***rige*** *”*, “da***cate*** *”*) and the other half corresponded to the Large Grapheme condition (e.g., “*ne* ***lain*** *”*, “*dan* ***eau*** *”*). Stimuli in all conditions were matched on orthographic Levenshtein distance 20^56^ and average bigram type and token frequencies using information from the Wuggy pseudo-word generator^57^.

In each trial, participants were presented in the centre of the screen with a 300 ms fixation cross, followed by the pseudo-word for 60 ms, and then a 100 ms mask. A question mark appeared on the screen following the mask and the participants were instructed to name the previously presented pseudo-word aloud as accurately as possible. The short timing of pseudo-word presentation aimed at allowing a single fixation, forcing rapid orthographic processing and avoiding ceiling responses. Verbal responses were recorded and coded for accuracy. As soon as the participant had responded the experimenter initiated the next trial. The order of trials was fully randomized and breaks were provided every 21 trials. Accuracy was analysed.

### General Procedure

Participants completed the questionnaire and then performed the behavioural tasks individually in a quiet, dimly lit room in a 45-minute session on the University campus. The order of tasks was counterbalanced between participants.

### Data Analyses

The VA span tasks were analysed using Type III ANOVAs (ULL R Toolbox^58^) including Group as a between-subject factor and relevant within-subject factors. Although normality assumptions were violated in these measures, particularly on letter positions on which performance was high, ANOVAs were used since they are robust to violations of normality; in cases of sphericity violations Greenhouse-Geisser corrections were used. The two groups were compared on other measures, such as chronological age and performance on the single letter identification task, using either parametric t-tests or, in cases of assumption violations, the non-parametric Wilcoxon signed-rank test. Accuracy scores by item and participant were available on the Pseudo-word Reading task and were thus analysed using generalized linear mixed effects models^59,60^ with the lme4 package^61^ using the logit link function and a binomial error distribution. Finally, one-tailed Pearson correlations between individual scores of a composite measure of VA span (computed from performance on the global and partial report reduced to the mean number of letters accurately processed in each trial^32^) and overall pseudo-word reading accuracy were performed within each group and for all participants. These correlations were performed after applying an exponential transformation to the VA span measures in order to improve their distribution and were one-tailed, based on the hypothesis of positive correlations between these measures. Other correlations, such as correlations between mean performance on the VA span tasks and performance on the single letter identification task, were performed using the non-parametric Kendall Tau-b correlation in cases of assumption violations. An alpha level of 0.05 was used for statistical tests and Hochberg corrections were applied to groups of post hoc comparisons and correlations.

### Data availability

The datasets generated during and/or analysed during the current study are available from the corresponding author on reasonable request.

## Electronic supplementary material

Supplementary Information
