# Supplementary material for: Enhancing reading performance through action video games: the role of visual attention span
Source: Sci Rep. 2017 Nov 6;7:14563. doi: 10.1038/s41598-017-15119-9 (PMC5674026; doi:10.1038/s41598-017-15119-9)
Supplement: Supplementary file 1 — Supplementary Information [file 41598_2017_15119_MOESM1_ESM.pdf]

Supplementary Material

Title: Enhancing reading performance through action video games: the role of visual attention span

Authors: Antzaka, A., Lallier, M., Meyer, S., Diard, J., Carreiras, M. & Valdois, S.

Supplementary Table S1: Percent correctly identified letters by Letter Position and Group (AVG and NVG) in Global Report.

Group		Position (% correct)					
		1	2	3	4	5	6
AVG (*n*=19)	Mean *(SD)*	98.46 *(2.85)*	98.68 *(1.99)*	97.59 *(3.2)*	88.16 *(8.36)*	76.54 *(9.44)*	89.04 *(10.13)*
	Range	91.67-100	95.83-100	91.67-100	62.5-100	58.33-87.5	66.67-87.5
NVG (*n*=17)	Mean *(SD)*	99.02 *(1.82)*	94.85 *(4.04)*	95.1 *(4.23)*	78.68 *(11.3)*	62.99 *(16.79)*	81.13 *(15.39)*
	Range	95.83-100	87.5-100	87.5-100	58.33-95.83	33.33-95.83	41.67-100

Supplementary Table S2: Percent correctly identified letters by Letter Position and Group (AVG and NVG) in Partial Report.

Group		Position (% correct)					
		1	2	3	4	5	6
AVG (*n*=19)	Mean *(SD)*	95.61 *(5.81)*	74.12 *(15.19)*	93.42 *(7.65)*	95.18 *(6.4)*	78.51 *(14.52)*	93.42 *(8.14)*
	Range	83.33-100	41.67-100	75-100	83.33-100	41.67-100	75-100
NVG (*n*=17)	Mean *(SD)*	82.84 *(22.34)*	66.18 *(24.73)*	91.67 *(18.63)*	90.2 *(20.88)*	69.12 *(24.07)*	78.43 *(15.88)*
	Range	33.33-100	8.33-91.67	25-100	16.67-100	8.33-100	41.67-100

Supplementary Table S3: Random and fixed effects coefficients for the model analysing pseudo-word reading accuracy (data points= 3022, items=84, subjects=36).

Group	Name	Random effects		
		Variance	*SD*	Correlation
Subject	Intercept	7.62E-01	8.73E-01	
	Grapheme Size	4.11E-02	2.03E-01	.24
Item	Intercept	7.39E-01	8.60E-01	

Group	Fixed effects			
	β	*SE*	*z*	*p*
Intercept	1.35E+00	1.81E-01	7.444	<0.001
Grapheme Size	7.34E-02	1.12E-01	0.655	.51
Word Presence	1.37E-01	1.06E-01	1.296	.19
Group	-3.68E-01	1.54E-01	-2.392	.017
Grapheme Size : Word Presence	8.25E-02	1.06E-01	0.781	.43
Grapheme Size : Group	2.11E-02	5.88E-02	0.359	.72
Word Presence: Group	-2.04E-02	4.71E-02	-0.432	.66
Grapheme Size : Word Presence: Group	-1.33E-02	4.71E-02	-0.283	.78

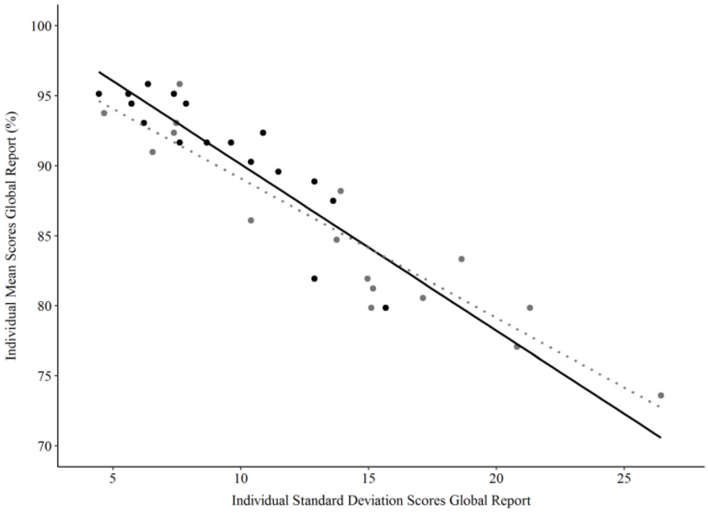

Supplementary Figure S1: Scatter plot depicting the negative correlation between individual standard deviation scores with individual mean % accuracy in Global Report for the AVG players (▬▬, *n*=19) and non-players (▪▪▪▪▪ ,*n*=17).

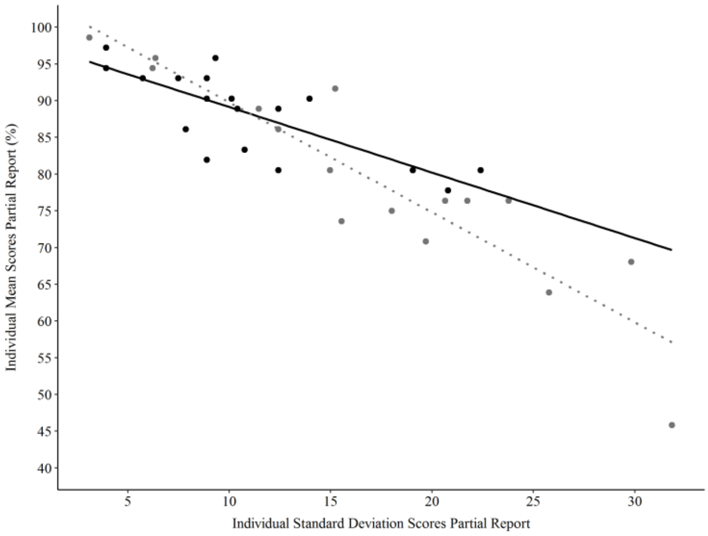

Supplementary Figure S2: Scatter plot depicting the negative correlation between individual standard deviation scores with individual mean % accuracy in Partial Report for the AVG players (▬▬ , *n*=19) and non-players (▪▪▪▪▪ ,*n*=17).

Supplementary Methods S1: Questionnaire on Language Background and Experience Playing Action Video Games

Nom : Prénom :

Date de naissance : ………………………………..

Langue maternelle : Français Autre : …………………

Autres langues parlées :

L2 : …………………….. courant avancé débutant

L3 : …………………….. courant avancé débutant

Apprentissage de la lecture en Français : OUI NON

Difficultés d'apprentissage de la lecture : OUI NON

Difficultés pour orthographier les mots* : OUI NON * *(indépendamment des accords)*

Que sont les jeux vidéo d'action ?

Les jeux vidéo d'action se caractérisent par :

- **La vitesse** : les objets apparaissent/disparaissent et se déplacent sur l'écran à grande vitesse.
- **La charge cognitive, perceptive et motrice élevée** : il faut traiter simultanément plusieurs informations et activer de nombreux plans moteurs en parallèle avant de prendre une décision.
- **L'emphase est mise sur le traitement visuel périphérique et l'attention divisée** : fréquemment les objets cibles apparaissent sur les bords de l'écran pendant que d'autres événements importants se produisent au centre de l'écran.

Quelques exemples de jeux vidéo d'action sont: first person shooter (Overwatch, Counter strike: Global Offensive, Titanfall 2, Splatoon, Battlefield 1), jeux de sport (FIFA 17 et NBA 2k17), jeux de plateforme (Super Mario Galaxy 2), real time strategy (League of Legends, DOTA 2) ….

Votre pratique des jeux vidéo d'action :

1. Jouez-vous aux jeux vidéo d'action ? OUI NON
2. Depuis combien de temps ? …………………….

Si vous êtes un joueur de jeux vidéo d'action, tournez la page et répondez aux questions suivantes….

Nous vous demandons de remplir le tableau ci-dessous. Pour cela…

Pensez aux 6 jeux vidéo d'action avec lesquels vous avez joué le plus dans la dernière année. Pour chaque jeu, écrivez le nom du jeu, le nombre d'heures que vous passez à y jouer par session, et le nombre de fois (sessions) où vous y jouez dans un mois. Indiquez aussi la console utilisée et si le jeu est un jeu vidéo d'action. Nous sommes seulement intéressés par les jeux auxquels vous avez joué au cours de l'année 2016.

Exemple 1. Si vous avez joué au Solitaire sur votre PC pendant environ 30 minutes à chaque session à raison de deux fois par semaine, vous pouvez écrire Solitaire (nom du jeu), 1/2 (heures par session), 8 (2 x 4 = 8 sessions par mois), PC (console) et Non (jeu vidéo d'action).

Exemple 2. Si vous avez joué au Final Fantasy XI sur votre Play Station 2 pendant des sessions d'environ 3 heures, une fois par mois, vous pouvez écrire Final Fantasy (nom du jeu), 3 (heures par session), 1 (sessions par mois), PS2 (console) et Oui (jeu vidéo d'action).

	Nom du jeu	Heures par session	Sessions par mois	Console	Jeu vidéo d'action
Ex. 1	Solitaire	1/2	8	PC	Non
Ex. 2	Final Fantasy XI	3	1	PS2	Oui
1					
2					
3					
4					
5					
6					

N'hésitez pas à rajouter des informations si nécessaire :

Supplementary Methods S2: Stimuli of Pseudo-word Reading task

Small Grapheme Size		Large Grapheme Size	
Word Present	Word Absent	Word Present	Word Absent
ri**mode**	nanoge	bi**rien**	ropien
na**pile**	siluve	ro**lion**	tiloix
si**cage**	vagase	né**lieu**	birieu
va**code**	ropume	na**coin**	lopuit
mé**cube**	todase	gi**bois**	pocain
pu**dame**	timoce	to**veau**	gufain
to**face**	vilibe	bu**pain**	nélain
ti**file**	covade	ru**beau**	daneau
so**gare**	porane	gi**main**	rutoin
vi**luge**	némude	po**gain**	vigeau
co**lave**	sorige	sa**foin**	fénien
ro**lime**	dacate	ti**loin**	saboux
pi**mine**	ritime	lu**gant**	tuvion
lo**muse**	pulale	vi**noix**	nuboin
fu**note**	fucuge	da**cuit**	luleau
li**puce**	lopise	lo**mien**	bugien
né**rage**	lifice	gu**peur**	nabant
po**ride**	pipode	tu**roux**	girion
da**tige**	mumige	fé**peau**	gidoin
ru**vase**	rucore	nu**pion**	rigois
mu**pose**	méfale	ri**bien**	toveur

Note: The embedded words are marked in bold
